# Stages of Grief Portrayed on the Internet: A Systematic Analysis and Critical Appraisal

**DOI:** 10.3389/fpsyg.2021.772696

**Published:** 2021-12-02

**Authors:** Kate Anne Avis, Margaret Stroebe, Henk Schut

**Affiliations:** ^1^Department of Clinical Psychology, Utrecht University, Utrecht, Netherlands; ^2^Department of Clinical Psychology and Experimental Psychopathology, University of Groningen, Groningen, Netherlands

**Keywords:** bereavement, stages of grief, Kübler-Ross, internet, websites, digital support, online resources, psychoeducation

## Abstract

Kübler-Ross’s stage model of grief, while still extremely popular and frequently accepted, has also elicited significant criticisms against its adoption as a guideline for grieving. Inaccurate portrayal of the model may lead to bereaved individuals feeling that they are grieving incorrectly. This may also result in ineffectual support from loved ones and healthcare professionals. These harmful consequences make the presentation of the five stages model an important area of concern. The Internet provides ample resources for accessing information about grief, raising questions about portrayal of the stages model on digital resources. We therefore conducted a systematic narrative review using Google to examine how Kübler-Ross’s five stages model is presented on the internet. We specifically examined the prominence of the model, whether warnings, limitations and criticisms are provided, and how positively the model is endorsed. A total of 72 websites were eligible for inclusion in the sample. Our analyses showed that 44 of these (61.1%) addressed the model, indicating its continued popularity. Evaluation scores were calculated to provide quantitative assessments of the extent to which the websites criticized and/or endorsed the model. Results indicated low criticalness of the model, with sites often neglecting evaluative commentary and including definitive statements of endorsement. We conclude that such presentation is misleading; a definitive and uncritical portrayal of the model may give the impression that experiencing the stages is the only way to grieve. This may have harmful consequences for bereaved persons. It may alienate those who do not relate to the model. Presentation of the model should be limited to acknowledging its historical significance, should include critical appraisal, and present contemporary alternative models which better-represent processes of grief and grieving.

## Introduction

The origin of the five stages model of grief can be traced to Kübler-Ross’s (1969) *On Death and Dying*. In this book, Kübler-Ross detailed her observations from interviews she conducted with patients who were dying of a terminal illness. Kübler-Ross’s fundamental premise was that the dying individual goes through five stages: denial, anger, bargaining, depression, and acceptance (often referred to by the acronym DABDA; in this article the five stages model). Already in this much-acclaimed volume, Kübler-Ross extended application of the five stages to the experience of (anticipatorily) bereaved persons, including a chapter to address *“The Patient’s Family.”* However, it was only decades later that Kübler-Ross, joined by Kübler-Ross and Kessler (2005), turned her attention specifically to bereavement in their 2005 book, *On Grief and Grieving*. This endeavor was to proceed subsequent to her death, with Kessler (2019) producing a third book, extending the model to include a sixth stage, namely, meaning. Looking back over more than four decades since *On Death and Dying* was published, it becomes evident that proponents as well as opponents of the five stages model have given credit to Kübler-Ross’s seminal work on death and dying, recognizing her enormous contribution in bringing these topics out into the open, to the benefit of many.

The five stages model of grief has been widely accepted by the general public, taught in educational institutions and used in clinical practice. To illustrate, widespread belief in the model was recently demonstrated in a survey conducted by Sawyer et al. (2021). When presented with the following statement “The process of grief can be expected to progress through a predictable series of stages, starting with denial and ending with acceptance,” as many as 30% of the general public believed this was definitely true, compared with 8% of mental health professionals, while an additional 38% of each of these groups answered that the statement was probably true. However, despite its popularity and recognition, various authors have criticized the model (for review: Stroebe M. et al., 2017). One issue often contested is the model’s representativeness of grief. This criticism stems from the fact that the model is based on interviews with terminally ill patients rather than bereaved individuals, making any claim that the five stages are an inevitability for bereaved people, unfounded. Over time, different authors have drawn attention to the ways that the model misrepresents the grieving process. For example, in their classic paper, “The Myths of Coping with Loss,” Wortman and Silver (1989) challenged the five stages model’s claim that all bereaved individuals will reach the final stage of acceptance. Furthermore, a study conducted by Bisconti et al. (2004) concluded that instead of a stage-like progression, emotional wellbeing appeared to oscillate back and forth following a loss. Corr (2019b) also drew attention to the variability of grief, warning against applying the stages of grief to all bereaved groups and highlighting the non-linearity of grief reactions. In her later work with David Kessler, Kübler-Ross herself appears to support the fluidity of grief, stating that the stages “are not stops on some linear timeline in grief. Not everyone goes through all of them or in a prescribed order” (Kübler-Ross and Kessler, 2005, p. 7). However, despite these subsequent cautions against using the stages in a rigid way, as Corr (2019b) points out, the use of the word “stages” in and of itself implies an orderly linear progression from one phase to another, which has resulted in many using the model as a prescriptive guideline rather than a descriptive model.

Such use of the model can also be harmful. Misutilization of the model may, for example, lead to grieving people feeling as if they are not grieving in the correct way and may result in ineffectual support from loved ones as well as from healthcare professionals. Echoing arguments voiced by others (e.g., Doka and Tucci, 2011; Konigsberg, 2011), Friedman and James (2008) offered the following conclusions to their own critique of the stages approach:

As much effort as we’ve put in to refuting the stages, Kubler-Ross herself rebuts them better than we can in the opening paragraph of On Grief and Grieving: “The stages have evolved since their introduction, and they have been very misunderstood over the past three decades. They were never meant to help tuck messy emotions into neat packages. They are responses to loss that many people have, but there is not a typical response to loss, as there is no typical loss. Our grief is as individual as our lives. Not everyone goes through all of them or goes in a prescribed order.”

If there are no typical responses to loss and no typical losses, and not everyone goes through them or in order, how can there possibly be stages that universally represent people’s reactions to loss? The fact is, no study has ever established that stages of grief actually exist, and what are defined as such can’t be called stages. Grief is the normal and natural emotional response to loss. Stage theories put grieving people in conflict with their emotional reactions to losses that affect them. No matter how much people want to create simple, iron clad guidelines for the human emotions of grief, there are no stages of grief that fit every person or relationship (p. 41).

Hall (2014) adds his understanding of the five stages appeal and cautions against oversimplicity:

Stage theories have a certain seductive appeal – they bring a sense of conceptual order to a complex process and offer the emotional promised land of “recovery” and “closure.” However, they are incapable of capturing the complexity, diversity and idiosyncratic quality of the grieving experience. Stage models do not address the multiplicity of physical, psychological, social, and spiritual needs experienced by bereaved people, their families and intimate networks. Since the birth of these theories, the notion of stages of grief has become deeply ingrained in our cultural and professional beliefs about loss. These models of grieving, albeit without any credible evidence base, have been routinely taught as part of the curriculum in medical schools and nursing programs (p. 8).

Given the criticisms summarized above, especially the possible harmful consequences relating to the misapplication of the five stages, the presentation of the model becomes an important concern. In a few recent analyses, Corr (2018, 2019a,^2020^) investigated this by exploring how the model was applied and criticized in a sample of American textbooks, in a sample of textbooks outside the United States, and in a sample of selected Social Work textbooks. These analyses indicated, amongst other things, the abiding popularity of the five stages, finding that they appeared in the majority of the sampled textbooks. Furthermore, while many authors included cautions about the model (most frequently stating that not everybody needs to experience all the stages or experience them in an orderly or fixed way), the model was often misrepresented. In Corr’s words:

Many authors of recent textbooks seen in this sampling have mischaracterized this theoretical model, most notably by failing to recognize its limitations, by not taking into account legitimate criticisms, and by running together an account of issues involved with dying with what they view as a broader accounts of dealing with loss and grief—and attributing that to *On Death and Dying* (Corr, 2018, p. 25).

While the analyses by Corr give insight into how the five stages are presented in textbooks, their presentation on the internet remains unexplored. As we become more reliant on technology, there are good reasons to argue that the internet will become an increasingly important resource for bereaved individuals to receive information concerning grief (including information about the five stages model): the internet provides a number of different types of resources for bereaved people including informational resources, internet forums, email groups, chat rooms, online memorial sites (Stroebe et al., 2008), internet therapy (Wagner et al., 2020), and social media sites (Moyer and Enck, 2020), with research indicating that bereaved persons make use of these digital resources (Vanderwerker and Prigerson, 2004; van der Houwen et al., 2010; Chapple and Ziebland, 2011). It seems important to examine the enduring influence of Kübler-Ross’s model through examination of its representation on internet sites.

The objective of this current study is, therefore, to examine how Kübler-Ross’s five stages model is presented on the internet. This was explored in a quantitative analysis addressing the following questions:

(a) How prominently and frequently is the five stages model mentioned on websites that provide information about grief following the death of a close person or pet?
(b) How is it judged? What warnings, limitations, and criticisms of the model are provided?
(c) How positively is this model endorsed?

In addition to exploring the above questions, we included finer-grained analyses. Evaluation scores based on the presence of warnings, limitations, criticisms, and endorsements of the five stages of grief are calculated for the websites. These scores enable a quantitative assessment of how critical and/or accepting the websites are of the five stages model.

## Materials and Methods

### Research Design

In this systematic narrative review, English and Dutch websites providing information about grief following the death of a close person or pet were selected using the search engine, Google. Data such as the type of website and the presence or absence of the five stages were extracted from the selected websites. Websites that mentioned the five stages model were further analyzed to determine what warnings, limitations, and criticisms concerning the model were provided and how the model was endorsed.

Evaluation scores were then calculated for the websites to provide a quantitative assessment of the extent that they criticized and endorsed the model. As there was no previously established scoring system available to evaluate the presentation of the stages on the internet, a novel one was developed by the authors of this study to calculate the scores. This scoring system can be found in Supplementary Data Sheet 1.

### Website and Text Excerpt Selection

A search was conducted in 2017 to select relevant websites. In 2020, the selected websites were revisited and data were extracted. The following search strings were used to find the websites: “grief,” “help with grief,” “how to deal with grief,” in Dutch “rouw,” “hulp bij rouw,” “hoe om te gaan met rouw.” These search strings were chosen, because in comparison to related words/phrases that the researchers considered (e.g., “bereavement” or “mourning”), these search strings corresponded to the most popular search terms in Google and, therefore, were expected to be words/phrases that most bereaved individuals struggling with the grief process would search for. In order not to restrict the search to the exact phrase match, search strings were entered into Google without quotation marks.

Websites were found *via* two search strategies. The first strategy involved typing the search terms directly into Google, using the three Google domains: ‘‘google.co.za,’’ ‘‘google.nl,’’ and ‘‘google.com.’’ This search strategy allowed researchers to access websites that individuals would have direct access to when searching from South Africa (google.co.za), Netherlands (google.nl) and worldwide (google.com)^1^. The choice to include the countries South Africa and Netherlands was made to ensure that both developing and non-English speaking regions were represented. To ensure adequate representation of these regions, a second search strategy was employed. In this strategy, a filter was applied in Google to ensure that only websites with South African (. co.za), Dutch (.nl), and generic domain extensions^2^ (. com/.org/.net) were accessed. For both search strategies, the first two pages of Google results were searched. The search was limited to the first two pages as these pages receive the most traffic and were, therefore, likely to capture websites accessible to the public when searching for information related to grief.

The websites were then examined to determine whether they mentioned the five stages model. Text excerpts on the websites that mentioned the model were analyzed to determine how the model was evaluated and endorsed. Only one text excerpt per website was analyzed, specifically the first excerpt found that mentioned the model. If a website did not mention the five stages model, only the website’s features (i.e., domain extension, type) were collected.

### Inclusion and Exclusion Criteria

The websites found using the search strategies were included if they: (a) provided information about the grief process following the death of a close person or pet (b) were written in English or Dutch. Exclusion criteria entailed websites with: (a) primarily audio or video content, (b) scientific research journals, (c) book chapters, (d) social media sites, (e) PDF files, and (f) dictionary definitions.

### Data Extraction

Data regarding the type of website, the website domain extension, and whether the website referred to the five stages model, were extracted by the first author of the study (KAA)^3^. The data for this study are provided in Supplementary Data Sheet 2. If the website mentioned the five stages model, the total word count of the text excerpt that mentioned the model, the word count of the description of the model (all information concerning the five stages model), and the word count of the description of DABDA (the description of the actual stages: denial, anger, bargaining, depression and acceptance) were also extracted.

Websites were categorized into the following different types based on their primary function: (a) service/product (selling a product or a service e.g., therapy sessions, funeral services, and books), (b) informational (providing knowledge, opinion or guidance about certain topics), (c) non-profit (dedicated to a particular cause or public benefit e.g., palliative care, mental health promotion), (d) religious (providing information and support from a religious perspective), and (e) news (devoted to national or global current events).

Websites that included the five stages model were then further analyzed by the same author to determine what warnings, limitations, criticisms, and endorsements of the model were provided (see Table 1 for definitions of these categories). The different warnings, limitations, criticisms and endorsements were determined using content analysis. Both a deductive and an inductive approach to content analysis was taken. Firstly, an a priori list of warnings, limitations, criticisms and endorsements was systematically compiled by the authors of the study based on close reading of the scientific and professional literature. Websites were then analyzed to determine additional warnings, limitations, criticisms, and endorsements. After analysis, the warnings, limitations, criticisms and endorsements were compared with each other for any similarities and dissimilarities to determine categorical and non-overlapping sub-categories to establish the final (sub)categorization system. The process to determine sub-categories was conducted by the first author (KAA) in consultation with the other two authors (MS and HS).

**TABLE 1:** Definitions warnings, limitations, criticisms, and endorsements of the five stages model.

	Definition
Warning	Putting one on guard against the five stages model by providing information regarding some caution or threat
Limitation	Pointing out that the five stages model is deficient in some quality
Criticism	A judgment of lack of merit of the five stages model, pointing out its faults, a censure
Endorsement	Showing support or approval of the five stages model

A random sample of 50 percent of the websites (22 websites) that mentioned the five stages model were then reanalyzed by the second author (MS) in order to assess interrater agreement concerning the different categories of warnings, limitations, criticisms, and endorsements of the model. To determine interrater agreement, a percentage was calculated by dividing the number of occasions raters agreed on the presence or absence of a category with the total possible agreements. Interrater agreement was established at 95.7 percent.

### Evaluation Scores

Evaluation scores were then calculated for the websites that mentioned the five stages. In order to calculate these scores, a scoring system was developed by the authors of this study (see Supplementary Data Sheet 1). To the best of our knowledge, no validated scoring system is available in the literature, to determine the level of criticalness and endorsement of a website. Thus, the authors developed their own system based on the warnings, limitations, criticisms, and endorsements found on the websites in this study. The first author (KAA) composed an initial version of the scoring system. This was then evaluated and revised by the other two authors (MS and HS). Thereafter, the system was piloted using a few websites to determine its feasibility. To ensure that the scoring system enabled detection of a website’s level of criticalness and endorsement, these pilot scores were then compared to the authors’ own general assessments of these attributes.

In this scoring system, the total score was determined by calculating (1) a score to determine how critical the website was of the five stages (based on the presence of warnings, limitations and criticisms) and (2) a score to determine how endorsing the website was (based on the presence of endorsements). As shown in the Supplementary Data Sheet 1, the score of endorsement was subtracted from the score of criticalness to determine a total score representing criticalness relative to endorsement, where the higher the score, the more critical the website was of the stages and the lower the score, the more endorsing it was.

## Results

### Website Characteristics, Prominence and Frequency of Inclusion of Five Stages Model

#### Sample Size

Eighty-three websites provided information about the grief process following the death of a close person or pet. However, 11 of these websites were excluded as the website link no longer worked when the data were analyzed in 2020 and therefore, the final sample size was 72.

#### Prominence and Frequency

Forty-four of the websites (61.1%) referred to the five stages model. Of the websites that did not specifically mention the five stages, nine mentioned the word “stages” without clear reference to which stage model they were referring to and, therefore, may have been referring to the five stages model. Additionally, results indicated that 27.9% of the total word count of all the text excerpts which mentioned the five stages was dedicated to describing and providing information about the five stages, with 15 of these excerpts (34.1%) allotting 50 percent or more of their total word count to the five stages model.

Table 2 shows the frequencies and percentages of the website domain extensions and types of websites in the total sample of websites, in the sub-sample of websites that referred to the five stages model and in the sub-sample of websites that did not refer to the model.

**TABLE 2:** Frequencies and percentages of website domain extensions and types in total sample and sub-samples where five stages model was absent and present.

		Total sample		Five stages		Five stages	
				present		absent	
		*n* = 72		*n* = 44		*n* = 28	
				
		*n*	%	*n*	%	*n*	%
**Domain extensions**							
	. com/.org/.net	31	43.1	22	50	9	32.1
	. nl	23	31.9	8	18.2	15	53.6
	. co.za	18	25	14	31.8	4	14.3
**Type**							
	Service/Product	26	36.1	16	36.4	10	35.7
	Informational	20	27.8	13	29.6	7	25
	Non-profit	14	19.4	8	18.2	6	21.4
	Religious	4	5.6	2	4.6	2	7.1
	News	8	11.1	5	11.4	3	10.7

In the total sample, 31 websites (43.1%) had a generic domain extension (. com/.org/.net), 23 websites (31.9%) had a Dutch domain extension (.nl) and 18 (25%) a South African domain extension (co.za). Notably, when considering the sub-sample of websites that did not mention the five stages model, 15 websites (53.6%) had Dutch domain extensions. In contrast, only eight websites (18.2%) that mentioned the five stages had a Dutch domain extension, with 22 websites (50%) in this sub-sample having a generic domain extension and 14 (31.8%) possessing South African domain extensions.

With regards to the types of websites, websites selling a service or a product were the most frequent type of website in the total sample (26, 36.1%), followed by informational (20, 27.8%), non-profit (14, 19.4%), news (8, 11.1%), and religious websites (4, 5.6%). A similar pattern was found in both the sub-samples of websites.

### Warnings, Limitations, and Criticisms of the Five Stages Model

To answer the question concerning how the five stages model was evaluated, the sub-sample of websites that referred to the five stages was analyzed to identify different warnings, limitations and criticisms.

Table 3 provides an overview of the frequencies and percentages of the different warnings, limitations and criticisms of the five stages model.

**TABLE 3:** Frequencies and percentages of warnings, limitations, and criticisms of five stages model.

		*N*	%
**Warning**			
	Warning: Non-rigidity		
	Non-linearity	26	59.1
	Not all 5 stages	22	50
	Varied intensity of stages	3	6.8
	No timetable/set time	12	27.3
	More than 5 stages	4	9.1
	Concurrency of stages	4	9.1
	Recurrence of stages	10	22.7
	Warning: Non-existence		
	Non-prescriptive	15	34.1
	Harmful	4	9.1
	Unhelpful	3	6.8
**Limitation**			
	Lack scientific research	4	9.1
**Criticisms**			
	Misapplied from terminal patients	4	9.1
	Other models superior	8	18.2
	Other metaphors superior	3	6.8
	Misrepresentation of grief	10	22.7

*The frequencies and percentages are based on the sub-sample of websites (n = 44) that referenced the five stages model.*

Two sub-categories of warnings were found on the websites. The first sub-category cautioned against the stages being taken in a rigid manner. This sub-category implied the existence of stages, but ascertained that the process whereby the stages are experienced can be different for each person or situation (e.g., the order of the stages or the time taken to complete the stages may differ). These types of warnings occurred often with the most frequent warning in this sub-category concerning the non-linearity of the five stages (26, 59.1%). Following this came warnings affirming that not all five stages have to be experienced (22, 50%), there is no set timetable or length of time for the stages to be completed (12, 27.3%), and that the stages could reoccur once completed (10, 22.7%). The second sub-category asserted that the five stages do not (always) exist. Non-prescriptive statements (i.e., wording that indicated that one does not need to experience the stages to heal) were the most common warning pertaining to this second sub-category (15, 34.1%).

When it came to the limitations and criticisms of the five stages model, statements that indicated that the five stages model does not represent the actual experience of grief (10, 22.7%) were most frequently mentioned. This was followed by wording suggesting the superiority of other models of grief (8, 18.2%). Other limitations and criticisms that were occasionally mentioned were: the lack of scientific research of the five stages model (4, 9.1%), the misapplication of the stages from the terminally ill (4, 9.1%), and the possible superiority of certain metaphors over the five stages (e.g., grief is a rollercoaster, 3, 6.8%).

### Endorsements of the Five Stages Model

The sub-sample of websites referring to the five stages model was further analyzed to gain insight into how the model was endorsed. The frequencies and percentages of these endorsements were calculated and are presented in Table 4.

**TABLE 4:** Frequencies and percentages of endorsements of five stages model.

		*N*	%
**Endorsement**			
	Endorsements: Non-definitive		
	Existence possible	7	15.9
	Helpfulness non-definitive	5	11.4
	Words of praise	8	18.2
	Word count DABDA > 30%	13	29.6
	Popularity	10	22.7
	Endorsements: Definitive/common		
	Existence common	14	31.8
	Existence definitive	22	50
	Existence definitive/non-rigid	18	40.9
	Helpfulness definitive	8	18.2

*The frequencies and percentages are based on the sub-sample of websites (n = 44) that referenced the five stages model.*

Statements of endorsements were found to fit into two sub-categories. The first sub-category contained statements that while endorsing of the stages, did not have a definitive essence. A description of the DABDA stages which covered more than 30 percent of the total word count of a website was the most common non-definitive endorsement (13, 29.6%). A relative word length criterion was included because it was reasoned that more extensive description draws more attention to the model, thereby highlighting the model’s importance and endorsing its existence. Thirty percent was chosen as a cut-off because, when examining the percentages of the word count describing DABDA versus total word count, two thirds fell below 30 percent, indicating that those above 30 percent were in the minority and, therefore, did not fit into the normal range of description.

**FIGURE 1:**
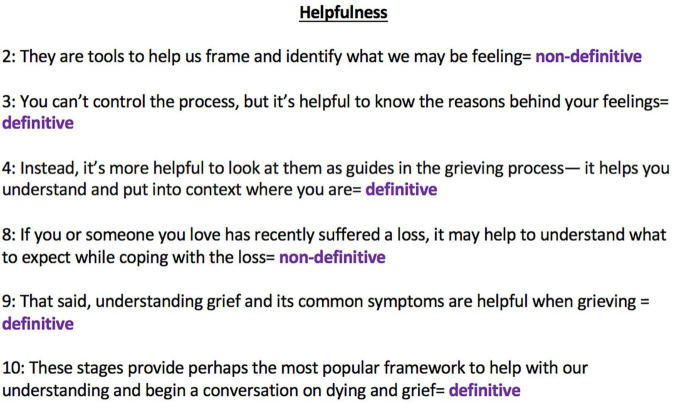
Examples of “helpfulness definitive” and “helpfulness non-definitive” statements. The numbers next to each statement correspond to the website ID from which the statements were taken.

The second sub-category pertained to statements that had a definitive nature or were close to definitive in nature (i.e., those suggesting that the stages were a common experience). These statements were frequently mentioned, with pure definitive statements about the existence of the five stages being the most common endorsement of the five stages model; 22 sites (50%) provided a statement that implied that the existence of the five stages was fact. Furthermore, 18 sites (40.9%) presented definitive statements about the existence of the five stages combined with a statement that this approach did not have to be followed in a rigid way (e.g., everyone will experience the stages, but you do not need to go through them in a specific order). Phrasing which indicated that the five stages were commonly experienced (e.g., most people, many people) was also a common endorsement of the five stages model (14, 31.8%), with definitive statements regarding the helpfulness of the five stages approach, occasionally mentioned (8, 18.2%; see Figure 1 for examples of definitive and non-definitive statements of helpfulness).

### Evaluation Scores

#### Determination of Scores

To determine a score of criticalness, points were assigned for the presence of warnings of existence, limitations and criticisms. In this system, warnings of rigidity were not included as a measure of criticalness because, on examination of these warnings, it became clear that they held low criticality toward the stages, cautioning only against the rigidity of the model, and thereby still endorsing the presence of the stages for all bereaved individuals.

Next, a score of endorsement was determined by assigning points for the different endorsements on the websites. The endorsement category “popularity” (statements highlighting the popularity of the stage approach e.g., well-known, popular, and famous) was not assigned points, as closer analysis showed that a number of sites that held a critical stance toward the stages also included statements of popularity but had few or no other endorsements. This called into question whether all statements of popularity could be viewed as endorsements of the five stages model, but rather as factual statements reflecting the stages’ widespread acceptance.

Lastly, a total score representing the level of criticalness relative to the level of endorsement was determined by inserting the separate scores of criticalness and endorsement into the following mathematical equation:


C⁢r⁢i⁢t⁢i⁢c⁢a⁢l⁢n⁢e⁢s⁢s⁢s⁢c⁢o⁢r⁢e-E⁢n⁢d⁢o⁢r⁢s⁢e⁢m⁢e⁢n⁢t⁢s⁢c⁢o⁢r⁢e=T⁢o⁢t⁢a⁢l⁢(h⁢i⁢g⁢h⁢e⁢r⁢i⁢s⁢m⁢o⁢r⁢e⁢c⁢r⁢i⁢t⁢i⁢c⁢a⁢l).

#### Summary of Results

Table 5 shows the means, medians and maximum and minimum scores for the separate and total scores of the measures of criticalness and endorsement. When determining the separate scores of criticalness and endorsement, the higher the score, the more critical or endorsing the website was of the stages (the highest possible score was 12), the lower the score, the less critical or endorsing it was (lowest possible score was zero). For the total score, the maximum possible score that could be obtained was 12 (indicating high criticalness) and the minimum score was −12 (indicating high endorsement), with a score of zero indicating a comparable level of endorsement and criticalness.

**TABLE 5:** Descriptive statistics for separate scores of criticalness and endorsement and total score of criticalness relative to endorsement.

		*M (SD)*	*Mdn*	Minimum	Maximum
**Score**					
	Criticalness	1.9 (3)	0	0	10
	Endorsement	3.6 (2.8)	3	0	10
	Total Score	−1.7 (4.9)	−2.5	−10	10

As indicated in Table 5, the mean score for the separate score of criticalness was 1.9 (*SD* = 3, *Mdn* = 0), while the mean score for the separate score of endorsement was 3.6 (*SD* = 2.8, *Mdn* = 3); almost double the mean score of criticalness. The mean total score of criticalness relative to endorsement was −1.7 (*SD* = 4.9) with a median score of −2.5. Taken together, these scores suggest overall low criticality of the websites toward the five stages model and a higher level of endorsement in relation to criticalness.

## Discussion

### Principal Findings

The purpose of this study was to gain better understanding of the presentation of Kübler-Ross’s five stages model on the internet. The concern to examine inclusion of the model on websites arose in large part from its critical assessment in scientific reviews and in the accounts of clinicians. Notably, scientific sources have drawn attention to the absence of a body of empirical research and lack of validity regarding the model. Clinicians have pointed to potential negative consequences for bereaved people who do not “conform” by going through the stages but who think that they should be experiencing them. In the face of these criticisms, it is important to explore how the model is presented to professionals and lay people in general, and to bereaved persons in particular. Technological advances have meant that the internet system is widely used for the giving to and seeking of support among bereaved persons, providing ample resources for accessing information about grief. This raises questions about the portrayal of the stages model through websites. We therefore conducted a systematic narrative review to examine the presentation of the five stages model of grief on the internet, investigating three research questions.

Our first research question addressed the prominence of the model; how frequently is it mentioned on websites providing information about grief? The results indicated the continued popularity of the model; 61.1% of websites included a description of the five stages, with accounts varying from brief mention to detailed elaboration of the model. This is a conservative estimate, given a further nine sites mentioned “stages” in general, indicating the possibility that nearly three quarters of all the sites referred to the model, at least non-specifically. This frequent inclusion is in line with Corr (2018, 2019a) research results; the five stages were described in the majority of his sampled textbooks. Similarly, it seems to echo Sawyer et al. (2021) findings mentioned earlier, that roughly 68% of the general public and 44% of mental health professionals endorsed the stages. Furthermore, an exploration of the word count providing information about the five stages also highlighted the prominence of the model, with over a third of the sites devoting 50% or more of their word count to the stages. Taken together, these results raise the question why there is such continued attention to the model, especially given that there have also been notable criticisms. The popularity of the model may stem from its ability to create order during a time of complexity, resulting in a positive narrative where one prevails over the despair of grief, culminating in the final stage of acceptance. The following quote cited on one of the reviewed websites encapsulates this: ‘Stage theories “impose order on chaos, offer predictability over uncertainty, and optimism over despair”’ (Shermer, 2008, p. 6). However, as the same website goes on to conclude, the appeal of the stages model in creating a narrative of hope is not equivalent to scientific importance: “Stages are stories that may be true for the storyteller, but that does not make them valid for the narrative known as science” (p. 9).

While our results showed that the five stages were mentioned frequently, closer examination of the data suggests differences in the portrayal of the stages between the included domain extensions. In particular, Dutch domain extensions appeared to refer to the model less frequently than the other domain extensions. This finding suggests that different countries may regard the model differently. The reasons behind these apparent differences are unclear, but one could speculate that a multitude of cultural and structural factors could play a role such as: underlying societal beliefs about death and dying, quality and quantity of educational programs providing information about issues surrounding grief, and ease of information accessibility, for example, to alternative models of grief.

Our second research question pertained to how the model was evaluated; what warnings, limitations and criticisms concerning the model were provided on the sites? Our exploration indicated that the most frequent types of warnings were those cautioning against the rigidity of the model, particularly nearly 60 percent of sites included warnings that the stages are non-linear and a half of the sites cautioned that not all five stages have to be experienced. This type of evaluation is also consistent with Corr (2018, 2019a) analyses, which established that non-linearity and not having to experience all stages were the most commonly mentioned critiques in his sample of textbooks. However, close examination of these types of warnings showed that they often lack a critical stance, endorsing the existence of the model by giving the message that one will/should experience the stages, just not in a rigid manner with the five stages following on in a strict order. Moreover, as critics have pointed out, the word “stages” itself implies rigidity, such that warning against rigidity actually presents a confusing message. This is one of the model’s most contentious features, with proponents using non-linearity to underline the model’s broad interpretation possibilities and therefore wider application, while opponents have argued that it disqualifies the model. Friedman (2009) made the latter point on one of the websites in our sample:

We [have] compared the stages of a butterfly to the alleged stages of grief, to show the problem with any stage theories of grief. To wit: Stages in order to be called stages must go through an orderly progression, each and every time. Starting as an egg, a potential butterfly must go through the four stages Egg, Caterpillar (Larva), Pupa (Chrysalis) Adult (Imago). It cannot elect to skip the larval stage and jump right over to the pupal stage.

Elisabeth Kübler-Ross herself constantly stated that the stages didn’t all happen and not necessarily in order, if at all. We just can’t find a way to use the idea of stages which really are absolute—see Butterfly reference—for something as variegated as human grief (p. 11).

In addition to warnings of rigidity, our analysis established that a number of warnings of existence, limitations and criticisms of the five stages model were sometimes included on some of the websites, albeit very infrequently (the mean score for criticalness was 1.9 out of a possible total score of twelve points). The fact that a large portion of websites lacked any critical appraisal highlights concerns about the representation of the model, particularly with regard to the lack of evidence and the potential for harm. These concerns should give one pause for reflection about the use of the five stages model as a contemporary guideline for bereaved.

Our final research question explored how the model was endorsed; how positively was it presented on the websites? Our analysis uncovered a number of different types of endorsements, which was defined in our study as statements showing support or approval of the five stages model. The most frequent endorsements were definitive statements (statements of unconditional approval) regarding the existence of the stages. As our results showed, the concerns we mentioned above were again confirmed. The high frequency of definitive statements about the stages’ existence is of considerable significance, since it suggests that the stages are an actuality; wrong conclusions about the validity of the five stages can easily be drawn by those accessing certain websites. The concern that this can have potentially harmful consequences for bereaved persons remains. The definitive endorsement of many sites on the internet can easily be interpreted as conveying the message that those who do not experience the stages are grieving incorrectly. As indicated earlier, advertising these stages as a certainty for bereaved people is unfounded. The implications of uncritical acceptance of the five stages model should not be underestimated; as one of the authors of our sampled websites cautions:

As we have pointed out in past articles, Kübler-Ross defined these “phases” as those experienced by a person dealing with the diagnosis of a terminal illness, and not as stages faced by someone who has faced a significant emotional loss. This misconception of their intended purpose has frustrated many grievers who felt that failure to progress through them could leave them forever in misery (Moeller, 2017, p. 6).

Furthermore, a definitive portrayal of the model can result in ineffectual support from loved ones or healthcare professionals. Insights from research on social and group norms have shown that violation of norms can lead to negative emotional reactions like anger or blame (Ohbuchi et al., 2004; Stamkou et al., 2019) as well as forms of social sanctions and punishment (Fehr and Fischbacher, 2004; Falk et al., 2005; Peters et al., 2017). A loved one or healthcare professional may, therefore, react in a negative way if they feel that the bereaved individual is violating the norm by not going through the stages. These reactions could result in bereaved people feeling alienated, an implication that is particularly worrying given that various studies have demonstrated the protective effect of social support in preventing negative effects in bereaved individuals (e.g., Hibberd et al., 2010; Çakar, 2020; Chen, 2020). Bereaved people themselves may also feel that there is something wrong with them for not grieving in line with the norm and may seek therapy to help move through the stages and grieve in the “correct” way. These endeavors may be unnecessary, especially considering that psychological interventions appear to be hardly or not effective for the bereaved population for whom there is no other indication (yet) than that they have lost a significant person (Schut et al., 2001; Wittouck et al., 2011). To put it concisely, presenting the five stages model in an uncritical and definitive light could lead to the belief that those who do not experience the stages are abnormal, a misconception which has important implications and potential harmful consequences for bereaved individuals.

In general, results showed low criticality with sites which often included definitive statements of endorsement neglecting such warnings. Our conclusion is that the model is not being accurately portrayed to bereaved people, with the dangers of using it as a contemporary guideline largely being ignored.

### Limitations of This Analysis

Limitations of this analysis need to be addressed. First, we noted the gap in time between the selection and analyses of the websites. While the majority of the sites were still operational when the data were analyzed (and, therefore, still relevant and accessible to the public as currently as 2020), an updated analysis could give insight into recent trends concerning the portrayal of the five stages model. This would be especially interesting in light of the recent corona pandemic. Many noteworthy questions have arisen regarding how the portrayal of grief has changed as a result of COVID-19, including ones about the application of theoretical approaches (cf., Stroebe and Schut, 2020). An analysis of information on grief-related websites subsequent to the current pandemic would add further insights into how understandings of grief have changed following COVID-19. For example, one relevant question in the context of our study is whether the sites have continued to advocate the five stages model under these changed circumstances.

Another limitation relates to the restriction to English and Dutch language websites. While this analysis ensured that both developing countries and non-English sites were represented, one avenue for future research could be to include more country-specific domain extensions, in order to achieve further representation of different cultures and languages and establish the influence of the five stages model in other parts of the world.

Additionally, an important limitation of this study has to do with the review process itself. Analysis of written text can lend itself to subjective interpretation (Given, 2008, p. 120–122). Certain warnings, limitations, critiques and endorsements were, for example, worded more implicitly than others, making them open to interpretation. An example of this is seen in the following text taken from one of our websites: “You may go back and forth between them or skip one or more stages altogether” (What is normal grieving WebMD, n.d., p. 4). While the text is not explicitly stating that the stages are non-linear, the phrase “back and forth” could be interpreted as implicitly implying non-linearity. An analysis of the researchers’ thought processes behind the determination of the different criticisms and endorsements revealed that while there was often agreement concerning the presence of a criticism or endorsement on a website (interrater agreement was nearly 96 percent), there was occasional disagreement about the exact statement representing these criticisms and endorsements. One possible explanation for this is that websites may possess multiple phrasing of the same premise, resulting in certain statements resonating with a particular individual more than others, but culminating in overall agreement of the message of the website. However, such differences occurred with too little frequency for the patterns of results to be affected.

Finally, two additional avenues for future research should be considered. Firstly, while the use of quantitative data was deemed appropriate for this study, future studies incorporating qualitative data may add additional insights (e.g., qualitative research may be better-able to establish whether the overall thrust of the five stages presentation in the website is endorsing, while only “lip service” is paid to criticisms). Furthermore, future research could play an important role in further validating the scoring system used in this study in the context of both digital and non-digital informational resources.

### Conclusion and Implications

Our analysis has revealed that the presentation of Kübler-Ross’s five stages model on websites raises a number of critical issues and implications, ones that need further consideration and which stand apart from her original contribution. Back in 1969, Kübler-Ross’s classic monograph *On Death and Dying* provided unprecedented (albeit anecdotal) insight into the process of adaptation among terminally ill people. The historical importance of her five stages model in bringing awareness to the experience of the dying cannot be denied. However, this historical impact does not mean that the model can be used as a contemporary standard for the grieving process. As some sites in our sample indicated, the model does not well-represent grief, lacks scientific evidence, and is potentially harmful. Reviewers have also drawn attention to the fact that it is purely descriptive, that it lacks explanatory power (e.g., to understand complications in grieving), and that there are better evidence-based alternatives (for reviews: Hall, 2014; Stroebe M.S. et al., 2017; Neimeyer, 2020). Researchers and clinicians alike have expressed strongly critical opinions. For example, researchers Silver and Wortman (2007) stated:

A mistaken belief in the stage model… can have devastating consequences. Not only can it lead bereaved persons to feel that they are not coping appropriately, but it also can result in ineffective support provision by members of their social network as well as unhelpful and potentially harmful responses by health care professional (p. 2692).

Grief counselors Friedman and James (2008) described “horror stories… heard from thousands of grieving people who’ve told us how they’d been harmed by them.”

It is improbable that authors of websites have the intention of causing harm to bereaved persons. In fact, the large number of resources, guidance and support many provide on their sites says otherwise. While some non-definitive statements of endorsement are unlikely to be harmful if provided together with a critical evaluation of the stages and especially in a historical context, a definitive, uncritical portrayal of the model may result in damaging consequences by alienating those who do not relate to the model. Authors of websites providing information about grief can still acknowledge the historical significance of the five stages model, while at the same time providing a critical appraisal of the stages to help prevent harmful consequences for bereaved people. The scoring system we developed for the purpose of this study could be used by authors (and perhaps readers) as a reference to assess the degree of critique/endorsement of the stages and the website’s quality.

It is our hope that our systematic narrative review of websites raises awareness to the potential dangers of presenting an uncritical view of the stages on the internet and provides authors with a guideline to help improve the information provided on their websites, ensuring that the shared goal of helping bereaved persons can be realized. Furthermore, we encourage the field to move beyond scholarly assessment and clinical experience of the model’s claims and premises, to acquire further verification of the harm done to bereaved people who are presented with the five stages as the way to grieve. This would strengthen the case for researchers and clinicians alike, supporting the effort to abandon the five stages model and turn to alternative, contemporary models of coping.

## Data Availability Statement

The original contributions presented in the study are included in the article/Supplementary Material, further inquiries can be directed to the corresponding author.

## Author Contributions

MS and HS conceived the original idea of the study. KA conducted the search for the websites and wrote the first draft of the article. KA and MS completed analysis of the websites. All authors developed the study design and scoring system and contributed to revisions and approved the final version before submission.

## Conflict of Interest

The authors declare that the research was conducted in the absence of any commercial or financial relationships that could be construed as a potential conflict of interest.

## Publisher’s Note

All claims expressed in this article are solely those of the authors and do not necessarily represent those of their affiliated organizations, or those of the publisher, the editors and the reviewers. Any product that may be evaluated in this article, or claim that may be made by its manufacturer, is not guaranteed or endorsed by the publisher.

## Footnotes

1 When the search for the websites was conducted, it was still possible to use different Google domains to access country-specific search results. A change at the end of 2017 had the effect that search results are now only relevant to the location the person is searching from, regardless of the Google domain used.

2 A domain extension is the last part of an URL, which represents the category or location of the website.

3 As indicated in the text, all three authors were involved in different parts of the data extraction process. The authors are all psychologists, researchers, and experts on grief and bereavement and are of mixed ages and genders.
