# Supplementary material for: Stages of Grief Portrayed on the Internet: A Systematic Analysis and Critical Appraisal
Source: Front Psychol. 2021 Dec 2;12:772696. doi: 10.3389/fpsyg.2021.772696 (PMC8675126; doi:10.3389/fpsyg.2021.772696)
Supplement: Supplementary file 1 [file Data_Sheet_1.docx]

**Scoring System**

**Explanation**

In the absence of alternative methods to evaluate the presentation of the five stages model of grief, this scoring system was empirically developed, adopting a strategy of content analysis to derive the categories. This scoring system determines how critical as well as how endorsing a website is of the five stages model by assigning points for the presence of warnings of existence, limitations, criticisms and endorsements.

In this system, two scores are calculated; one score representing criticalness and one score representing endorsement. The endorsement score is then subtracted from the criticalness score to determine a total overall score; the higher the score the more critical the website is of the stages, the lower the score, the more endorsing.

**Score 1: Criticalness**

To determine the criticalness of the five stages model, assign the below points for the presence of warnings of existence, limitations and criticisms. The higher the score, the more critical the website is of the five stages model, the lower the score, the less critical.

**Note:** The definitions for the different warnings, limitations and criticisms can be found at the end of this scoring system.

| ***Warnings*** | |
| --- | --- |
| *+1 point* | 1 warning regarding existence of stages present |
| *+2 points* | 2 warnings regarding existence present |
| *+3 points* | 3 warnings regarding existence present |
| ***Criticisms and Limitations*** | |
| *+2 points* | 1 criticism or limitation present |
| *+4 points* | 2 criticisms and/or limitations present |
| *+6 points* | 3 criticisms and/or limitations present |
| *+9 points* | 4 or more criticisms and/or limitations present |

**Maximum Score (high criticalness):**

**12** (=3 warnings + 4 or more criticisms/limitations present)

**Minimum Score (low criticalness):**

**0** (=0 warnings, criticisms/limitations present)

**Score 2: Endorsement**

To determine the level of endorsement of the five stages model, assign the below points for the presence of endorsements. The higher the score, the more endorsing the website is of the five stages, the lower the score, the less endorsing.

**Note:** The definitions for the different endorsements can be found at the end of this scoring system.

| ***Endorsements: Non-definitive*** | |
| --- | --- |
| *+1 point* | 1 non-definitive endorsement present |
| *+2 points* | 2 non-definitive endorsements present |
| *+3 points* | 3 non-definitive endorsements present |
| *+4 points* | 4 non-definitive endorsements present |
| ***Endorsements: Definitive/Common*** | |
| *+2 points* | 1 definitive endorsement present |
| *+4 points* | 2 definitive endorsements present |
| *+6 points* | 3 definitive endorsements present |
| *+8 points* | 4 definitive endorsements present |

**Maximum Score (high endorsement):**

**12** (=4 non-definitive+ 4 definitive endorsements present)

**Minimum Score (low endorsement):**

**0** (=0 endorsements present)

**Total Score: Criticalness relative to Endorsement**

To determine the total overall score for criticalness relative to endorsement, insert the separate scores for criticalness and endorsement into the below equation. The higher the score, the more critical the website is of the five stages model, the lower the score, the more endorsing. A score of zero signifies a comparable level of criticalness and endorsement.

| Equation: |
| --- |
| $\boldsymbol{Criticalness Score-Endorsement Score}$  $\boldsymbol{= Total Score (higher is more critical)}$ |
|  |
| Maximum Score (high criticalness) = 12 |
| Minimum Score (high endorsement) = -12 |

**Definitions: Warnings of Existence, Limitations, Criticisms and Endorsements**

| **Warnings:**  **Existence** |  |
| --- | --- |
|  |  |
| Non-prescriptive: | Wording that implies that you do not have to experience the stages to heal e.g. there is no typical loss, this is just one approach, stage approach is wrong. |
| Harmful: | Indication given that five stages can be harmful in some way e.g. they impose guilt, pressure & doubt, they can leave you feeling like you are not grieving well. |
| Unhelpful: | Statement that the stage approach is not (always) helpful. |
|  |  |
| **Limitations** |  |
|  |  |
| Lack scientific research: | Statement given that there is no (reliable) evidence that supports the five stages. |
|  |  |
| **Criticisms** |  |
|  |  |
| Misapplied from terminal patients: | Statement provided to imply the misapplication of stages from the terminally ill to the bereaved e.g. based on dying patients not bereaved, improper shift to stages of grief. |
| Other metaphors superior: | Worded in a way to suggest that there are other metaphors that may be/are superior to the stage approach e.g. grief may feel more like a rollercoaster. |
| Other models superior: | Worded in way to suggest that other models may be/are superior to the stage approach. |
| Misrepresentation of grief: | Some statement given that stage model does not represent actual experience of grief e.g. grief is more complex, grief does not occur in stages. |
|  |  |
| **Endorsements:**  **Non-definitive** |  |
|  |  |
| Existence possible | Wording indicating that the stages are a possibility e.g. your feelings may happen in stages as you come to terms with your loss. |
| Helpfulness non-definitive | Statement is given indicating that the five stages model can/may be helpful e.g. stages can be helpful for some people. |
| Words of praise | Positive aspect of stage approach is provided (not helpfulness or popularity) e.g. well respected, wise. |
| Word count DABDA > 30% | Word count of text giving description of DABDA is more than 30 percent of the total word count.  **Note:** this only concerns the word count of the description of the actual stages i.e. word count describing denial, anger, bargaining, depression and anger, not the additional text providing contextual information about the stages e.g. historical information about the stages, criticisms etc. |
|  |  |
| **Definitive/Common** |  |
|  |  |
| Existence common | Wording indicating that stages are commonly experienced e.g. most/many people will experience the stages. |
| Existence definitive | Definitive statement about existence of stages i.e. statement of unconditional approval e.g. you will go through the stages when experiencing a loss, there are five stages of grief. |
| Existence definitive/non-rigid | Definitive statement about existence of stages, but same statement also suggests that stages do not have to be experienced in a rigid way e.g. you will experience the five stages, but not in exact order. |
| Helpfulness definitive | Definitive statement given regarding the helpfulness of the stages e.g. the stage approach is helpful to understand grief. |
